# Association between miR499b A>G gene polymorphism and recurrent pregnancy loss in Turkish women

**DOI:** 10.1590/1806-9282.20242027

**Published:** 2025-08-08

**Authors:** Ulas Coban, Esra Tekcan, Sengul Tural

**Affiliations:** 1 Ondokuz Mayıs University, Faculty of Medicine, Department of Obstetrics and Gynecology – Samsun, Turkey.; 2 Ondokuz Mayıs University, Faculty of Medicine, Central Laboratory – Samsun, Turkey.; 3 Ondokuz Mayıs University, Faculty of Medicine, Department of Medical Biology – Samsun, Turkey.

**Keywords:** Gene polymorphism, Abortion, Habitual, microRNA, Polymorphism, Single nucleotide

## Abstract

**OBJECTIVE:**

Recurrent pregnancy loss is characterized as a disorder characterized by two or more miscarriages, and its etiology is unclear. Recent research has focused on microRNA and recurrent pregnancy loss; however, single-nucleotide polymorphisms of microRNA in recurrent pregnancy loss need to be better understood. The aim of the study was to assess if there is any relationship between the miR499b A>G gene polymorphism and recurrent pregnancy loss in Turkish women.

**METHODS:**

The work has 267 participants, including 153 patients in the recurrent pregnancy loss and 114 participants in the control group. DNA isolation from peripheral blood was conducted using a kit-based approach, followed by the implementation of polymerase chain reaction and restriction fragment length polymorphism methods as outlined in the procedure.

**RESULTS:**

The rates of recurrent pregnancy loss were determined as 32.7% for the AA genotype, 51.6% for the AG genotype, and 15.7% for the GG genotype. When the AA genotype type was taken as a reference, the risk of recurrent pregnancy loss was 0.922 times higher (95%CI 0.513–1.655; p=0.785) in the AG type and 0.354 times lower (95%CI 0.178–0.705; p=0.003) in the GG type. In the recurrent pregnancy loss patient group, the GG genotype was lower than the expected value. The GG genotype was found to be less susceptible to recurrent pregnancy loss development.

**CONCLUSION:**

Based on our results, GG genotype frequencies, which are the recessive model of the miR499b A>G gene polymorphism, may be a protective genotype in susceptibility to recurrent pregnancy loss in Turkish women. The results obtained from this study represent the first data to be established for the Turkish population.

## INTRODUCTION

A pregnancy loss is the spontaneous termination of a pregnancy before the fetus attains viability, affecting around 15–40% of all pregnancies^1^. Most sporadic miscarriages before the 10th week of gestation are due to chromosomal abnormalities involving numerical alterations, particularly trisomy, monosomy, and polyploidy. Recurrent pregnancy loss (RPL) is defined as a condition characterized by two or more unsuccessful clinical pregnancies. Though its etiology is still not completely understood, several factors are believed to influence RPL. Research on risk factors of RPL has investigated various aspects, including lifestyle factors like smoking, maternal obesity, and alcohol consumption, and others like age, antiphospholipid syndrome, uterine anomalies, thrombophilia, hormonal or metabolic problems, infections, autoimmune disorders, sperm quality, and genetics^1,2^. Based on the commonly accepted perspective, the chance of experiencing another pregnancy loss goes up with each previous miscarriage and surpasses 50% after 5–6 occurrences^3^. Most published sources indicate that analyzing genetic factors and determining appropriate treatment options is promising in RPL^3^. While increased RPL susceptibility was associated with specific gene variants, only a few of these genes were confirmed to contribute to RPL pathogenesis^4^. However, clinical genetic testing is still not advanced and lacks a comprehensive molecular investigation component. These molecular investigations can aid in a better insight into the mechanisms driving RPL and treatment options in the future^2^.

After the 1960s, a specific group of ribonucleic acid (RNA) molecules was discovered regulating the process of gene expression by regulating the activation or inhibition of particular genes. They are called "non-coding" because they do not contain the genetic information necessary to make a protein. These non-coding RNAs are separated into several groups depending on their functions and where they are found. One of the most researched classes of short non-coding RNAs is microRNAs (miRNAs)^5^. miRNAs are short non-coding RNAs (22–23 nucleotides) that adjust actions after transcription. miRNAs have been shown to control about 60% of all human genes that code proteins and are involved in numerous physiological mechanisms and states of diseases^4^. miRNAs are crucial in determining cellular fate by regulating cell development, maturation, differentiation, apoptosis, cell signaling, cellular interactions, homeostasis, and angiogenesis. Single-nucleotide polymorphisms (SNPs) occurring in miRNA sequences have the potential to impact their regulatory function by changing their expression. SNPs show their effect by making alterations in the seed region of miRNA that can modify the interaction strength between miRNA and their mRNA targets. Second, SNPs can influence the maturation of pre-miRNA into its active, functional form. Finally, miR polymorphisms can modify the epigenetic regulation of miRNA genes^6^.

In studies conducted so far, miRNAs have been associated with many diseases^3,7^. Although lots of different types of miRNAs have been investigated in different regions, the literature is insufficient on the *miR499b* A>G Gene (rs10061133) polymorphism and RPL. In the present study, we planned to explore the association between the *miR499b* A>G gene (rs10061133) polymorphism and RPL in Turkish women.

## METHODS

### Study sample

Between the years 2023 and 2024, those who applied to the Department of Obstetrics and Gynecology at Ondokuz Mayıs University were provided written consent and a questionnaire to gather information on various factors, including age, the number of miscarriages, the number of pregnancies, hormone levels, and other conditions like thyroid diseases, other endocrine disorders, gynecological anomalies, hypertension, systemic diseases, smoking and alcohol history, and polycystic ovary syndrome were questioned. Recurrent miscarriage was defined as a history of two or more pregnancy losses for the patients, while the control group consisted of women who had experienced uncomplicated pregnancies without any history of pregnancy loss. The subjects who had any diseases or conditions other than RPL, as mentioned above, were excluded from the sample group. Participants were of Turkish descent, sharing a common ethno-geographic origin. A sum of 267 women who expressed interest in the study were distributed into two groups. The RPL group consists of 153 women, and the control group consists of 114 women. A volume of 2 mL of peripheral venous blood was drawn from all participants and transferred into an ethylenediaminetetraacetic acid (EDTA) tube. After labeling, the tubes were kept in storage at −20°C. Genomic deoxyribonucleic acid (DNA) isolation of individuals was performed from peripheral blood. For DNA isolation, the Qiagen DNA Isolation Kit was used. The effect of the *miR499b* A>G allele polymorphism on RPL was compared between these groups.

### Deoxyribonucleic acid isolation and polymerase chain reaction and restriction fragment length polymorphism methods for *miR449b* A>G polymorphic region

The polymerase chain reaction and restriction fragment length polymorphism (PCR-RFLP) method was used for determining the *miR449b* A>G polymorphic region. In the PCR, primer pairs were used with a final concentration of 0.6 picomol/μL. PCR reaction was performed by completing the total volume to 25 μL with ddH_2_O. GGT ATC CAG AGC ACT TCA TTG ACA-3′ and 5′-ACC TGA ATC AGG TAG GCA GTG TCT-3′ primers were used for the PCR technique. Temperature conditions in PCR: Denaturation at 95°C for 5 min, 35 cycles as denaturation at 95°C for 1 min, hybridization at 60°C for 1 min, elongation at 72°C for 30 s, and final extension at 72°C for 7 min. After PCR, the PCR products were loaded on a 2% agarose gel with 5 μL and checked in agarose gel electrophoresis. If amplification of the correct gene region was observed, restriction endonuclease was used. BsmAI enzyme was used for polymorphic region detection in amplified 119 bp PCR products (New England BioLabs, Ipswich, MA, USA). Restriction enzyme cleavage results were evaluated on 3% agarose gel. AA type (119-bp), AG type (119-bp, 97-bp, and 22-bp), and GG type (97-bp and 22-bp) were evaluated.

### Statistical analysis

The data were analyzed using IBM Statistical Package for the Social Sciences (SPSS) V23. Differences in allele and genotype frequencies between RPL cases and controls were compared using logistic regression and the chi-square test, respectively. Odds ratios (ORs) and 95% confidence intervals (CIs) were used to assess the strength of the correlation between genotypes and RPL. p<0.05 level was established as significant.

## RESULTS

The mean age of all participants was 32.01±5.73 years. The clinical and demographic findings of the groups are shown in Table 1.

**Table 1:** Demographic characteristics and clinical variables of recurrent pregnancy loss patients and control subjects.

Characteristics	Controls subjects (n=114)	RPL patients (n=153)	p-value
Age, year	31.50±5.32	32.39±6.00	0.33
FSH	8.11±1.98	7.91±2.22	0.37
LH	4.57±1.74	4.97±1.72	0.02*
E2	30.39±8.38	32.74±9.19	0.01*

Values are presented as mean±standard deviation. FSH: follicle-stimulating hormone, LH: luteinizing hormone, E2: estrogen; RPL: recurrent pregnancy loss.

(*) Asterisk indicates statistical significance (p<0.05).

**Figure 1:**
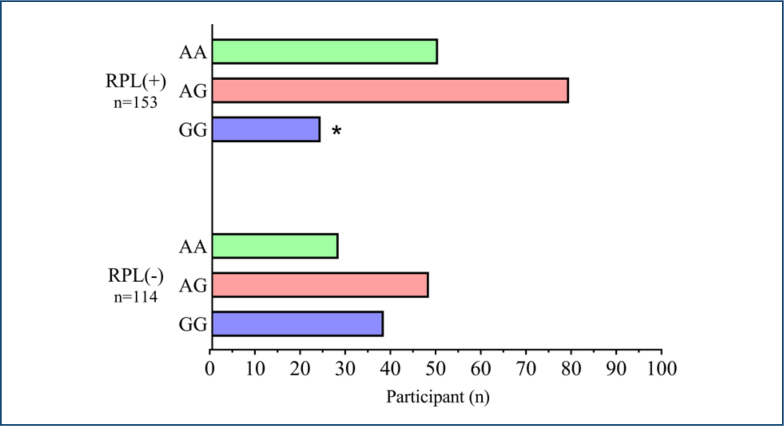
Distribution of genotype frequencies among recurrent pregnancy loss patients and control group. Asterisk (*) indicates statistical significance (p<0.05).

The rates of RPL were determined as 32.7% for the AA genotype, 51.6% for the AG genotype, and 15.7% for the GG genotype (Table 2). When the AA genotype type was taken as a reference, the risk of RPL was found to be 0.922-fold higher in the AG type (95%CI 0.513–1.655; p=0.785) and 0.354-fold lower in the GG type (95%CI 0.178–0.705; p=0.003) (Figure 1).

**Table 2:** Genotype frequencies of the *miR499b* A>G polymorphism between controls and recurrent pregnancy loss patients.

Genotypes		Control subjects (n=114)	RPL patients (n=153)	OR	95%CI	p-value
miR-423b A>G rs10061133						
	AA	28 (24.6%)	50 (32.7%)	1.000 (reference)		
	AG	48 (42.1%)	79 (51.6%)	0.922	0.513–1.655	0.785
	GG	38 (33.3%)	24 (15.7%)	0.354	0.178–0.705	**0.003** *
Dominant (AA vs. AG+GG)				0.671	0.389–1.156	0.149
Recessive (AA+AG vs. GG)				0.372	0.207–0.667	**<0.001** *
Allele						
	A	104	179	1.7	1.188, 2.376	**0.001** *
	G	124	127			

RPL: recurrent pregnancy loss; OR: odds ratio, CI: confidence interval, p<0.05 is statistically significant.

(*) Asterisk indicates statistical significance (p<0.05).

Bold formatting is used to highlight statistically significant values.

## DISCUSSION

In this investigation, we found that a rise in the G allele in miR449b is less sensitive in predisposing Turkish women to the development of RPL. The GG genotype was observed at a lower frequency in RPL patients compared to healthy controls. In recent years, there have been many genetic studies revealing the relationships between RPL and genetic polymorphisms, and still, there are unclear points in the etiopathogenesis of unexplained RPL^6,8,9^.

miRNA polymorphism may alter the receptivity of the endometrium and affect implantation failure, leading to recurrent abortion. The receptivity of the endometrial stroma is one of the vital levels for trophoblast invasion and placental advancement through a pregnancy outcome. The pre-miRNA SNP miR-499b A>G has been reported to have positive associations with different types of carcinomas, in which case it might exert a considerable influence on RPL through cell proliferation^10^.

Studies have demonstrated the significant involvement of miRNAs at the cellular and molecular levels in the maintenance of pregnancy^6^. Histologically, the placenta comprises trophoblast, endothelial, and mesenchymal cells, whose activities, including proliferation, differentiation, invasion, decidualization, and angiogenesis, collectively contribute to a healthy pregnancy process^11^. Since studies have shown that the pregnancy process is influenced by genetic, environmental, and physiological factors, miRNAs must strike a delicate balance and play a pivotal role in this equilibrium^11,12^. miRNAs are being suggested as potential risk factors for RPL, and there is an increasing interest in exploring the influence of gene-environment interactions on RPL^8,13^.

Alipour et al. conducted a study in Iran with miRNA polymorphism and RPL. They found a positive correlation between the *miR499a* A>G polymorphism, the *miR499* G allele polymorphism, and increased susceptibility to RPL^14^. Considering its mechanism, the *SOX6* gene is a direct target of the *miR499* gene and suppresses the expression of *FGF-3*, which takes part in cell proliferation and differentiation during embryonic development^6,9^. Also, another study by Parveen et al. in Northern India revealed that the *miR499a* A>G polymorphism might be a predisposition for RPL. They found that the mi499b AG+GG genotype was tied to a higher risk than AA^15^. But unlike these studies, Amin et al. from Iran found no noteworthy differences for *miR499a* genotype and allele frequencies between women in cases and controls^6^. Also, Jeon et al. found that *miR499* AG+GG and the combination of *miR196a2CC*/*miR499* AG+GG were significantly associated with idiopathic RPL in Korean women^8^. Fazli et al. from Korea found a significant elevation in the prevalence of the *miR499* gene polymorphism AA/AG+GG and GG/AG+AA^10^.

In the literature, there are few studies investigating the relationship between the *miR499b* (rs10061133) polymorphism and RPL. Rah et al. studied the susceptibility of RPL to *miR499b* from Korea and found that GG and GG+AA alleles have an increased risk, which is different from our study^16^. Kim et al. found an association between only the AG genotype of the *miR499b* (A>G) polymorphism and the risk of RPL when compared with the AA genotype.

Conversely, no statistically significant associations with the risk of RPL were observed in the case of other genotypes, including both dominant and recessive genotype models^17^. Diverse regional factors might contribute to the varying pathogenesis of RPL and its connection to SNPs in miRNA^18^. Babker et al. in their recent RPL and polymorphism studies in Sudanese women showed an association between *Human Platelet Antigen-1* (*HPA-1*), *Human Platelet Antigen-3* (*HPA-3*), *Factor XIII* gene polymorphism, and RPL^19^. They also suggested that *ACE I/D* polymorphism is strongly associated with unexplained spontaneous abortion and that *ACEI/D* polymorphism increases pregnancy complications. They predicted that Sudanese women may experience spontaneous abortion due to *ACE I/D* polymorphism^20^. In another study, the research findings suggested that *MTHFR C677T*, *FVL G1691A*, and *FII G20210A* variants do not significantly contribute to increased susceptibility to RPL in Sudanese women^21^.

Identifying the targets that cause RPL formation is the fundamental step to eliminate these targets. Therefore, it is of great importance to increase the scope and continuity of genetic and epigenetic scientific research to develop more detailed and personalized strategies and thus improve women's reproductive health.

In previous studies also, *FSHR*, *HRG*, *TP73*, and *VEGF* gene variations were investigated with ovarian response, and statistically significant differences were detected^22–24^. It is recommended to implement new methods and techniques in this study group^25–28^.

## CONCLUSION

In this study, we evaluated the potential association between the *miR499b* A>G gene polymorphism and susceptibility to RPL in the Turkish population. As a result of our findings, it is thought that GG genotype frequencies, which is the recessive model of the *miR499b* A>G gene polymorphism, may be a protective genotype in susceptibility to RPL development. The GG genotype was found to be less susceptible to RPL development. In sick individuals, the GG genotype was lower than the expected value. Therefore, the GG genotype may be a protective genotype in proximity to RPL development. So, comparing the results with those of other populations can give us new information about how environmental and genetic differences affect RPL.

The findings of this study would provide a valuable contribution to the existing literature on this topic and also in establishing biomarkers for RPL for diagnosis and treatment in the future. However, a cohort study with a larger number of participants is needed to better understand the association between specific genes and RPL and the mechanism of the association so that these genes can be used for diagnosis.

### Limitations

Increasing the sample size and diversity of ethnic groups in the study will increase the precision and accuracy of the study. The results can be supported by including epigenetic studies and showing the effects of polymorphisms in the *mir499b* gene with changes in the *mir499b* expression level.

## Data Availability

The datasets generated and/or analyzed during the current study are available from the corresponding author upon reasonable request.
